# Upgrading the Repertoire of miRNAs in Gastric Adenocarcinoma to Provide a New Resource for Biomarker Discovery

**DOI:** 10.3390/ijms20225697

**Published:** 2019-11-14

**Authors:** Michelle E. Pewarchuk, Mateus C. Barros-Filho, Brenda C. Minatel, David E. Cohn, Florian Guisier, Adam P. Sage, Erin A. Marshall, Greg L. Stewart, Leigha D. Rock, Cathie Garnis, Wan L. Lam

**Affiliations:** 1 Department of Integrative Oncology, British Columbia Cancer Research Centre, Vancouver, BC V5Z 1L3, Canada; mpewarchuk@bccrc.ca (M.E.P.); bminatel@bccrc.ca (B.C.M.); dcohn@bccrc.ca (D.E.C.); fguisier@bccrc.ca (F.G.); asage@bccrc.ca (A.P.S.); emarshall@bccrc.ca (E.A.M.); gstewart@bccrc.ca (G.L.S.); lrock@bccrc.ca (L.D.R.); cgarnis@bccrc.ca (C.G.); wanlam@bccrc.ca (W.L.L.); 2 International Research Center, A.C.Camargo Cancer Center, Sao Paulo 01508-010, Brazil; 3 Department of Pulmonology, Thoracic Oncology and Respiratory Intensive Care & CIC-CRB INSERM 1404, Rouen University Hospital, 76000 Rouen, France; 4 QuantIF-LITIS EA 4108, IRIB, Rouen University, 76000 Rouen, France; 5 Faculty of Dentistry, Dalhousie University, Halifax, NS B3H 4R2, Canada; 6 Department of Surgery, Division of Otolaryngology, University of British Columbia, Vancouver, BC V5Z 1L3, Canada

**Keywords:** gastric cancer, novel microRNAs, prognostic factors, noncoding RNAs

## Abstract

Recent studies have uncovered microRNAs (miRNAs) that have been overlooked in early genomic explorations, which show remarkable tissue- and context-specific expression. Here, we aim to identify and characterize previously unannotated miRNAs expressed in gastric adenocarcinoma (GA). Raw small RNA-sequencing data were analyzed using the miRMaster platform to predict and quantify previously unannotated miRNAs. A discovery cohort of 475 gastric samples (434 GA and 41 adjacent nonmalignant samples), collected by The Cancer Genome Atlas (TCGA), were evaluated. Candidate miRNAs were similarly assessed in an independent cohort of 25 gastric samples. We discovered 170 previously unannotated miRNA candidates expressed in gastric tissues. The expression of these novel miRNAs was highly specific to the gastric samples, 143 of which were significantly deregulated between tumor and nonmalignant contexts (*p*-adjusted < 0.05; fold change > 1.5). Multivariate survival analyses showed that the combined expression of one previously annotated miRNA and two novel miRNA candidates was significantly predictive of patient outcome. Further, the expression of these three miRNAs was able to stratify patients into three distinct prognostic groups (*p* = 0.00003). These novel miRNAs were also present in the independent cohort (43 sequences detected in both cohorts). Our findings uncover novel miRNA transcripts in gastric tissues that may have implications in the biology and management of gastric adenocarcinoma.

## 1. Introduction

Within the last five years, large-scale, whole-genome profiling efforts by groups such as The Cancer Genome Atlas (TCGA) consortium have provided an invaluable genetic repository that has enabled the discovery of many cancer-driving genes [1]. These initiatives have led to the first definition of the gastric adenocarcinoma (GA) microRNA (miRNA) transcriptome and uncovered important regulatory roles for these noncoding transcripts [1]. While many genetic and epigenetic mechanisms are known to result in miRNA deregulation in cancer, GA-specific miRNA deregulation networks have also been implicated in disease biology [2]. Indeed, the frequent disruption of miRNAs has been associated with malignant transformation in gastric epithelial cells [3,4]. Clinically, the expression levels of certain miRNAs have been characterized as noninvasive diagnostic biomarkers that may inform chemotherapy resistance and patient prognosis [5,6,7].

The algorithm that is most commonly employed in the discovery of miRNAs is miRDeep2, which does so using secondary structural features and consistency with Dicer processing [8]. Initial miRNA-identification projects that have dominated the miRNA literature, including publicly accessible databases such as miRBase, emphasize abundant and conserved sequences [9]. There has been a recent push to identify previously unannotated miRNAs that are highly organ-specific, which has expanded the known miRNA transcriptome [9,10,11,12]. Similar to past miRNA-identification projects, these organ-specific analyses rely upon algorithms such as miRDeep2, but they do not concern themselves with sequence conservation between different tissues, and impose less stringent cut-offs on the expression level, which has allowed them to discover miRNAs missed by earlier investigations [9,10,11,12]. The discovery of these novel organ-specific miRNAs has prompted us to reevaluate the raw-sequence data generated by TCGA to identify novel miRNAs in order to upgrade the GA transcriptome and build a new resource for miRNA-based biomarker and therapeutic discovery in this difficult-to-treat cancer.

## 2. Results

### 2.1. Discovery of Novel miRNA Sequences in Gastric Tumor and Nonsalignant Samples

**Figure 1:**
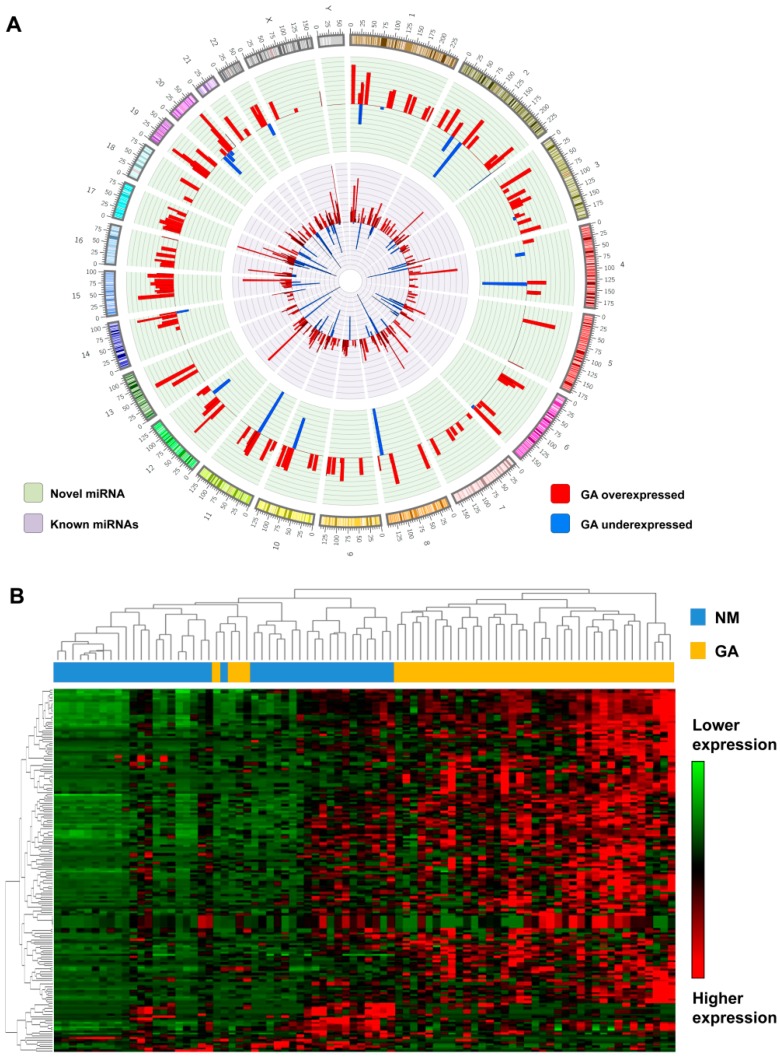
Updated microRNA (miRNA) expression patterns in gastric adenocarcinoma. (**A**) Genomic distribution of novel and known miRNAs identified in gastric tissues and their respective expression levels (log2 tumor/nonmalignant ratio). This circular illustration of the genomic localization of the novel and known miRNAs was created using Clico (https://cgdv-upload.persistent.co.in/cgdv/). The histogram represents the fold change values (log2) from the miRNA expression in gastric adenocarcinoma (GA) compared to nonmalignant samples (bar widths of miRNA genomic position were adjusted to 10 MB for illustration). (**B**) Unsupervised hierarchical clustering analysis of the expression of 170 novel miRNAs shows a clear separation of tumors (yellow) from nonmalignant samples (blue; matched cases from 41 patients).

Using small-RNA sequencing from TCGA (Table 1), we detected the expression of 505 known miRNAs (previously annotated in miRBase v.22) and similarly identified 170 novel miRNA candidates (without overlap with miRBase-annotated miRNAs). Molecular characteristics shared between novel candidates and annotated miRNA, including predicted secondary structure, nucleotide composition, and genomic distribution with known miRNAs, support their identity as true miRNAs (Figure 1A).

Among the 170 novel miRNAs, 85 had detectable expression in both malignant and nonmalignant samples, with the remaining specifically expressed in one context (70 miRNAs only in malignant and 15 in nonmalignant samples). The unsupervised hierarchical clustering of novel miRNA expression demonstrated a clear distinction between GA and nonmalignant samples (Figure 1B).

### 2.2. Context and Tissue-Specific Expression Patterns of Novel miRNAs

Most of the novel miRNAs (143 of 170, or 84%) were differentially expressed (*p*-adjusted < 0.05) in GA compared to nonmalignant samples, of which the majority showed a relatively higher expression in tumor samples (132 overexpressed and 11 underexpressed; Supplemental Table S1). This expression pattern parallels that observed in the known miRNAs, where most were differentially expressed (366 of 505, or 72%), and the majority displayed increased expression in GA relative to nonmalignant samples (333 overexpressed; 33 underexpressed; Supplemental Table S2).

To determine whether the novel miRNAs exhibit organ-specific expression patterns, we examined the 100 novel miRNAs that were detected in nonmalignant stomach samples. In a t-distributed stochastic neighbor embedding (t-SNE) dimensionality-reduction analysis, their expression pattern clearly distinguished gastric samples from those of other organs available in TCGA, including liver, pancreas, and lung samples (Supplemental Figure S1).

### 2.3. MicroRNA Prognostic Score to Predict Gastric Adenocarcinoma Patient Outcome

**Figure 2:**
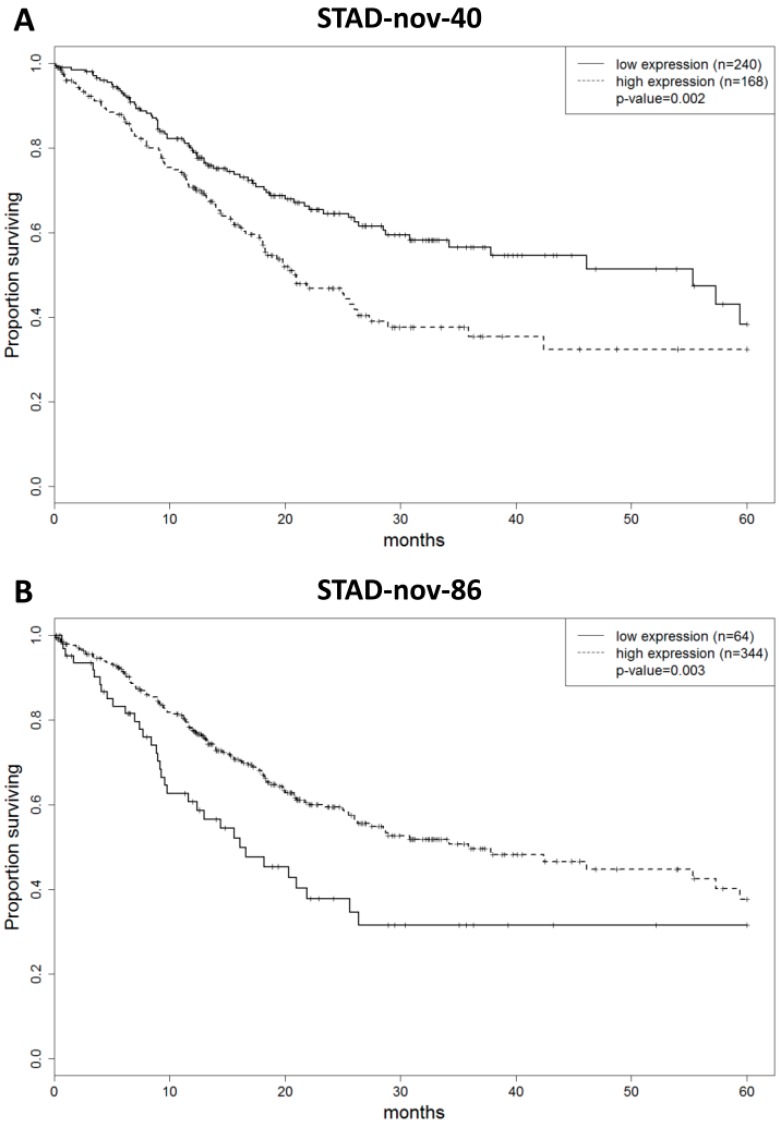

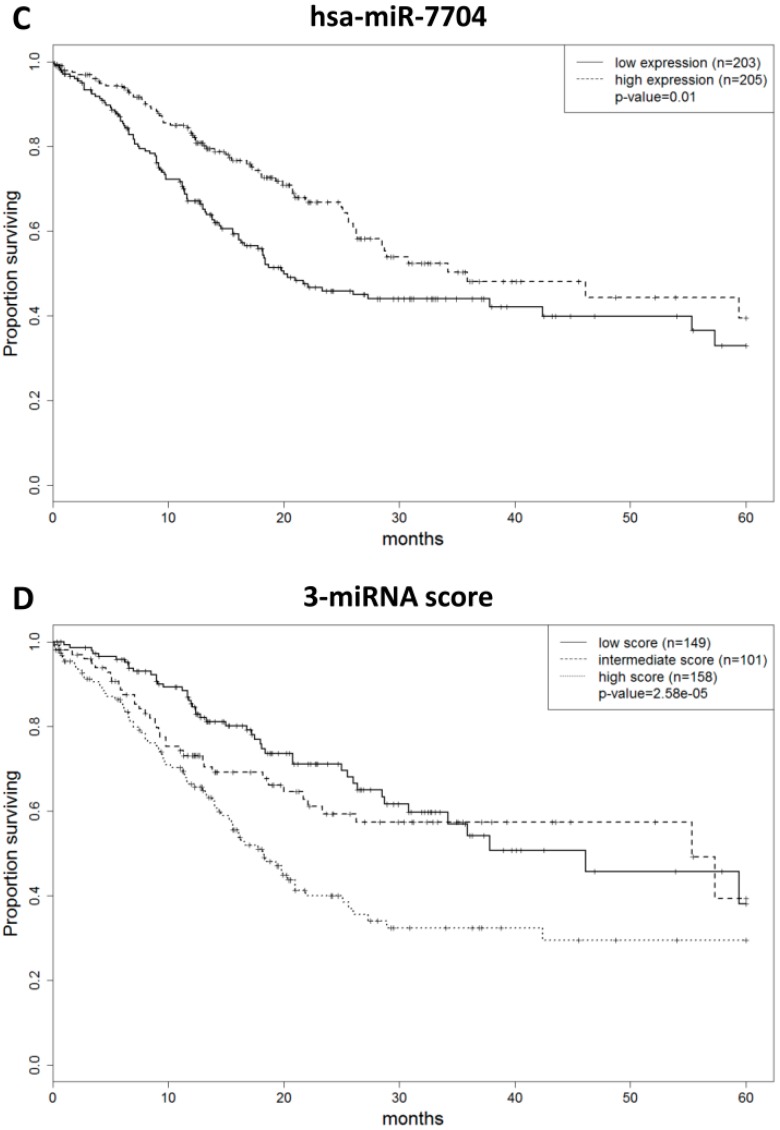
Expression of known and novel miRNAs is predictive of gastric adenocarcinoma patient outcome. The plots show the overall survival of GA patients from TCGA (*n* = 408), according to (**A**) STAD-nov-40, (**B**) STAD-nov-86, (**C**) hsa-miR-7704, and the (**D**) combined expression of STAD-nov-40, STAD-nov-86, and hsa-miR-7704.

The prognostic value of known and novel miRNAs expressed in gastric tumors was assessed in the TCGA dataset. In a univariate analysis, 75 annotated and nine novel miRNAs were significantly associated with overall survival. Multivariate analysis showed that two novel miRNAs (STAD-nov-40 and STAD-nov-86) and one annotated miRNA (hsa-miR-7704) were independently associated with overall survival (*p* < 0.01). A higher expression of STAD-nov-40 and lower expression of STAD-nov-86 and hsa-miR-7704 were related to shortened survival (Figure 2A–C). Using a combined score of these three miRNAs (prognostic score = STAD-nov-40 × 0.657 − STAD-nov-86 × 0.663 − hsa-miR-7704 × 0.825), we were able to segregate patients into three prognostic subgroups (Figure 2D; *p* = 2.6 × 10^5^). The median survival was 46.1 months for patients with low scores (n = 149; CI95% (30.8-not reached)), 55.3 months for patients with intermediate scores (n = 101; CI95% (23.3-not reached)), and 18.3 months for patients with high scores (n = 158; CI95% (15.5–25.1)) (Supplemental Table S3). Notably, prognostic novel miRNAs (STAD-nov-40 and STAD-nov-86) were found to be expressed in an independent cohort of gastric samples. Despite the limited sample size of this second cohort (19 gastric tumor and six nonmalignant gastric tissue samples), 43 of the novel miRNAs were also found to be expressed. Patient survival information was unavailable in this dataset for prognostic evaluation. Twenty-five mRNAs were predicted as targets of at least two of the three miRNAs comprising the prognostic score, playing potential roles in hypoxia inducible factor 1 signaling (*p*-adjusted = 0.037), small cell lung cancer (*p*-adjusted = 0.041), Hepatitis B (*p*-adjusted = 0.042), and cyclic adenosine monophosphate signaling (*p*-adjusted = 0.049).

## 3. Discussion

Here, we discover 170 new miRNAs in gastric cancer, representing an expansion of the gastric miRNA transcriptome by more than one-third. We determine that these miRNAs are able to distinguish gastric tissue from other tissue types, suggesting their potential use as tissue-of-origin markers, as gastric cancer is known to metastasize to a variety of organs (Supplemental Figure S1). We confirm the expression of 43 of these miRNAs in a validation cohort, in spite of that cohort’s restricted size. A larger validation cohort would likely allow confirmation of additional miRNAs.

We find that 84% of these newly discovered miRNAs are differentially expressed between cancer and nonmalignant tissues, with over 90% of those differentially expressed miRNAs being more highly expressed in GA. This suggests that these miRNAs may play a part in the development and regulation of key tumor phenotypes, which, in turn, indicates a potential role as prognostic markers. Indeed, a measure that combines the expression of two novel miRNAs with one known miRNA (hsa-miR-7704) is an independent predictor of the overall survival of GA patients. The group of mRNAs predicted to be targets of two or more of these three miRNAs is enriched for those involved in a number of different pathways, including HIF-1 signaling and cAMP signaling. Both HIF-1 signaling [13,14] and cAMP signaling [15] have previously been linked to poor outcomes in gastric cancer, likely due to an increase in the invasiveness of gastric cancer cells. Interestingly, the lower expression of hsa-miR-7704 is already known to be associated with more advanced stages of gastric cancer and a higher risk of liver metastasis in rectal cancer [16,17]. These results are particularly encouraging in light of the numerous studies describing the potential of noncoding RNA species to predict tumor aggressiveness and patient outcome [3].

The dysregulation of miRNA expression during the natural history of gastric adenocarcinoma has previously been characterized [4]. Using a miRNA microarray platform in malignant and precursor gastric lesions, 42 miRBase-annotated miRNAs were reported as altered in early gastric adenocarcinoma. Eight miRNAs from this list were not human or were removed from the current miRBase database, nine did not pass our detection threshold criteria, and from the remaining 25 miRNAs, 11 were identified as differentially expressed in GA by both studies (hsa-miR-18a, hsa-mir-34c, hsa-miR-96, hsa-miR-145, hsa-miR-200a, hsa-miR-370, hsa-miR-410, hsa-miR-589, hsa-miR-651, hsa-mir-654, and hsa-miR-655).

Our discovery has substantially expanded the group of miRNAs known to be expressed in gastric adenocarcinoma. The failure of previous studies to characterize these novel miRNAs suggests that, unlike previously known miRNAs, these novel transcripts are not highly conserved between tissues. This is supported by our finding that the expression pattern of these novel miRNAs is capable of distinguishing gastric samples from other tissues. Hence, these novel miRNAs may hold substantial clinical utility as tissue-of-origin biomarkers in cases of advanced metastases with unknown primaries. Additionally, the ability of novel miRNA expression to stratify patients by outcome suggests that incorporating novel miRNAs into current gene-based diagnostic panels may improve their predictive capacity.

While the function of these previously unannotated sequences remains to be characterized in vitro, our search for miRNA sequences expressed in gastric tissue uncovered novel, gastric-specific miRNAs. Our work demonstrates that previous attempts at miRNA characterization have overlooked less abundant and tissue-specific miRNAs. We find that these new sequences have prognostic value and are able to identify patients with good and poor survival in gastric adenocarcinoma. This work expands upon the known gastric miRNA transcriptome and provides a valuable resource for biomarker and therapeutic target discovery.

## 4. Materials and Methods

### 4.1. Data Processing and miRNA Discovery

**Figure 3:**
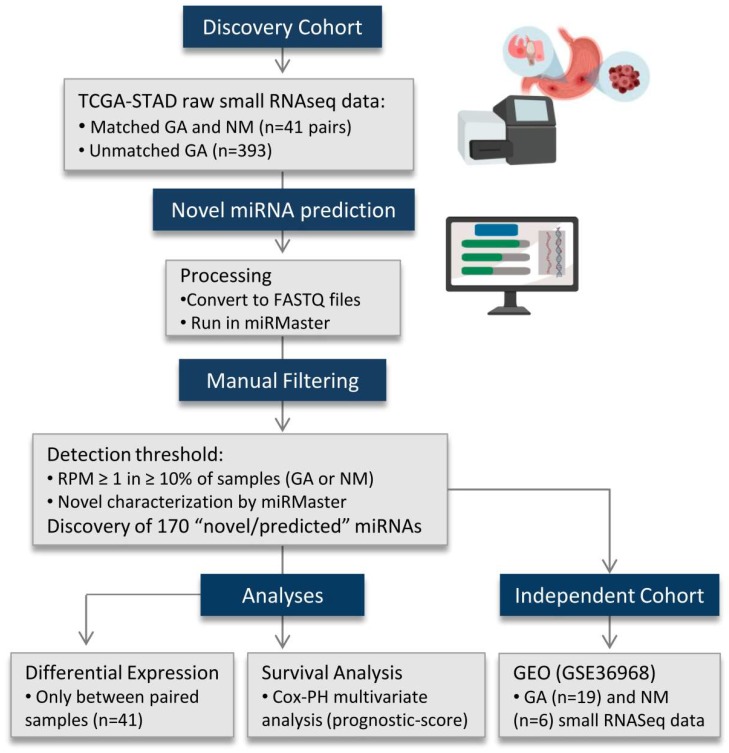
Flow diagram outlining the major steps of the study. Novel miRNAs were predicted using the miRMaster tool on small RNA sequencing data from the TCGA stomach cohort. One-hundred and seventy novel miRNA candidates were curated based on their expression in non-malignant and malignant samples. These sequences showed a global overexpression in gastric tumors when compared to non-malignant samples. A second, publicly available, small RNA sequencing cohort (GSE36968: 19 tumors and six non-malignant gastric samples) was assessed. Forty-three novel miRNA candidates were detected in both the discovery and independent cohorts. The novel miRNAs were submitted to differential expression and overall survival analyses (univariate and multivariate analyses). GA: Gastric adenocarcinoma; NM: Non-malignant; RPM: Reads per million; COX-PH: Cox proportional-hazards.

Small RNA sequencing data from GA (*n* = 434, Figure 3, Table 1) and adjacent non-malignant stomach tissue (*n* = 41) were obtained from The Cancer Genome Atlas (TCGA, https://cancergenome.nih.gov/; GDC Project ID: 6208). Clinical data were obtained through UCSC Xena (http://xena.ucsc.edu/). Binary Alignment Map (BAM) files were converted to unaligned FASTQ files (PartekFlow, Partek, Inc). Sequencing data from a second cohort of non-malignant (*n* = 6) and tumor (*n* = 19) gastric samples retrieved from the Gene Expression Omnibus (GSE36968, http://www.ncbi.nlm.nih.gov/gds) were also examined.

Unaligned small RNA sequencing data from TCGA were processed using miRMaster (https://ccb-compute.cs.uni-saarland.de/mirmaster). Using this platform, we performed quality filtering and aligned the resulting reads to the hg38 build of the human genome. We then predicted putative novel miRNAs using an algorithm analogous to miRDeep2, a well-established and widely applied [9,18,19] novel miRNA discovery tool that predicts novel sequences [20]. Candidate miRNAs were filtered to include only sequences with a detectable expression of ≥1 read per million (RPM) in ≥10% of samples, for each group. The same expression cut-off was applied for the known miRNAs annotated in miRBase (version 22) [21]. The existence of the discovered sequences was verified in a secondary cohort (GSE36968) based on their genomic loci. Putative novel miRNA showing 10 or more raw reads among all GSE36968 samples were considered expressed.

### 4.2. Assessment of Tissue and Context Specificity of the Novel miRNAs

The combined expression patterns of the novel miRNAs detected in the non-malignant samples of our discovery dataset were also queried in 456 additional non-malignant samples from TCGA (bile duct, bladder, brain, cervix, colon, head and neck, kidney, liver, lung, pancreas, prostate, stomach, and thyroid). The cross-tissue expression of these candidates was assessed using non-linear t-distributed stochastic neighbor embedding (t-SNE). Additionally, an unsupervised hierarchical clustering analysis (Pearson correlation and complete linkage metrics) was performed on the 170 novel miRNAs in the 41 paired gastric adenocarcinoma and non-malignant samples.

The differential expression of miRNA candidates was determined by a paired *t*-test with Benjamini–Hochberg correction (*p*-adjusted < 0.05; fold change > 1.5) comparing the miRNA expression (RPM values were not log-transformed) of 41 GA and non-malignant samples derived from the same patients (RStudio v.3.5.1).

### 4.3. Development of an miRNA-Prognostic Score

Novel and known miRNAs (155 and 494, respectively) detected in tumor samples (≥1 RPM in at least 10% of GAs) were assessed for their associations with patient outcome in the TCGA cohort using a univariate log-rank analysis (*p* < 0.05). Follow-up information was available for 408 of the 434 total patients (http://xena.ucsc.edu/).

MicroRNA sequences that had significant associations with overall survival (9 novel and 75 known miRNAs) were then included in a multivariate Cox proportional hazard model. miRNAs significantly associated with overall survival (*p* < 0.01) were combined using the Cox model coefficients to define a prognostic miRNA score, which was subsequently assessed using a log-rank test.

### 4.4. Target Prediction and Pathway Enrichment Analysis

Target prediction of the novel (STAD-nov-40 and STAD-nov-86) and known miRNA comprising the prognostic model (hsa-miR-7704) was performed using the miRanda v3.3 algorithm [22] against all human gene 3’untranslated regions (3′UTR) (alignment score ≥ 160; total energy ≤ −20 kcal/mol; total score ≥ 160). The mRNA targets of at least two of the three miRNAs were subject to in-silico pathway analysis using the pathDIP tool, using experimentally detected and computationally predicted protein–protein interactions in the Kyoto Encyclopedia of Genes and Genomes (KEGG) database (*p*-value BH-adjusted < 0.05) [23].

## Figures and Tables

**Table 1:** Summarized clinical data from the 434 gastric adenocarcinoma patients from The Cancer Genome Atlas (TCGA).

Features ^1^	Number (%)
Gender	
Male	280 (64.5)
Female	154 (35.5)
Age	
Median	67
≥55	362 (83.4)
<55	69 (15.9)
N/A	3
Tumor Histologic Grade	
G1	10 (2.3)
G2	155 (35.7)
G3	260 (59.9)
GX	9 (2.1)
Distant Metastasis (M)	
M0	382 (88)
M1	30 (6.9)
MX	22 (5.1)
Regional Lymph Nodes (N)	
N0	129 (29.7)
N1	116 (26.7)
N2	85 (19.6)
N3	86 (19.8)
NX	17 (3.9)
Discrepancy	1 (0.2)
Primary Tumor (T)	
T1	23 (5.3)
T2	91 (21)
T3	192 (44.2)
T4	118 (27.2)
TX	10 (2.3)
Stage	
I	58 (13.4)
II	128 (29.5)
III	182 (41.9)
IV	42 (9.7)
N/A	16 (3.7)
Discrepancy	8 (1.8)
H. pylori	
Yes	19 (4.4)
No	161 (37.1)
N/A	254 (58.5)

^1^ The clinical data were collected from UCSC Xena (http://xena.ucsc.edu/). The samples were a part of the TCGA-STAD dataset; N/A: Not available.

## Supplementary Materials

Supplementary materials can be found at https://www.mdpi.com/1422-0067/20/22/5697/s1.

## Abbreviations

BAM	Binary Alignment Map
BH	Benjamini–Hochberg correction
CI95	95% Confidence interval
GA	Gastric adenocarcinoma
GEO	Gene Expression Omnibus
KEGG	Kyoto Encyclopedia of Genes and Genomes
miRNA	microRNA
NM	Adjacent non-malignant tissue
RPM	Read per million
sncRNAs	Small non-coding RNAs
TCGA	The Cancer Genome Atlas
t-SNE	t-Distributed stochastic neighbor embedding
UTR	Untranslated regions
