# Supplementary material for: Upgrading the Repertoire of miRNAs in Gastric Adenocarcinoma to Provide a New Resource for Biomarker Discovery
Source: Int J Mol Sci. 2019 Nov 14;20(22):5697. doi: 10.3390/ijms20225697 (PMC6888638; doi:10.3390/ijms20225697)
Supplement: Supplementary file 1 [file ijms-20-05697-s001.zip › Supplemental_Figure.docx]

**Figure S1.** Demonstration of tissue-specificity of the predicted novel miRNAs identified in non-malignant gastric tissue. The t-SNE analysis was performed with 456 non-malignant samples collected from TCGA in bile duct, bladder, brain, cervix, colon, head and neck, kidney, liver, lung, pancreas, prostate, stomach, and thyroid tissues. It was performed using normalized expression levels derived from the 100 predicted novel miRNAs identified in non-malignant gastric tissue.

| 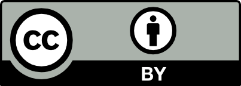 | © 2019 by the authors. Submitted for possible open access publication under the terms and conditions of the Creative Commons Attribution (CC BY) license (http://creativecommons.org/licenses/by/4.0/). |
| --- | --- |
